# Supplementary material for: Direct and Label-Free Monitoring of Albumin in 2D Fatty Liver Disease Model Using Plasmonic Nanogratings
Source: Nanomaterials (Basel). 2020 Dec 15;10(12):2520. doi: 10.3390/nano10122520 (PMC7765559; doi:10.3390/nano10122520)
Supplement: Supplementary file 1 [file nanomaterials-10-02520-s001.pdf]

Supplementary Materials

Direct and Label-Free Monitoring of Albumin in 2D Fatty Liver Disease Model Using Plasmonic Nanogratings

Gerardo A. Lopez-Muñoz [1], Ma. Alejandra Ortega [1], Ainhoa Ferret-Miñana [1], Francesco De Chiara [1] and Javier Ramón-Azcón [1,2,*]

[1] Institute for Bioengineering of Catalonia (IBEC), The Barcelona Institute of Science and Technology, Baldiri I Reixac, 10-12, 08028 Barcelona, Spain; glopez@ibecbarcelona.eu (G.A.L.-M.); mortega@ibecbarcelona.eu (M.A.O.); aferret@ibecbarcelona.eu (A.F.-M.); fdechiara@ibecbarcelona.eu (F.D.C.)

[2] ICREA-Institució Catalana de Recerca i Estudis Avançats, 08010 Barcelona, Spain

* Correspondence: jramon@ibecbarcelona.eu; Tel.: +34-934-039-735

Supporting Information

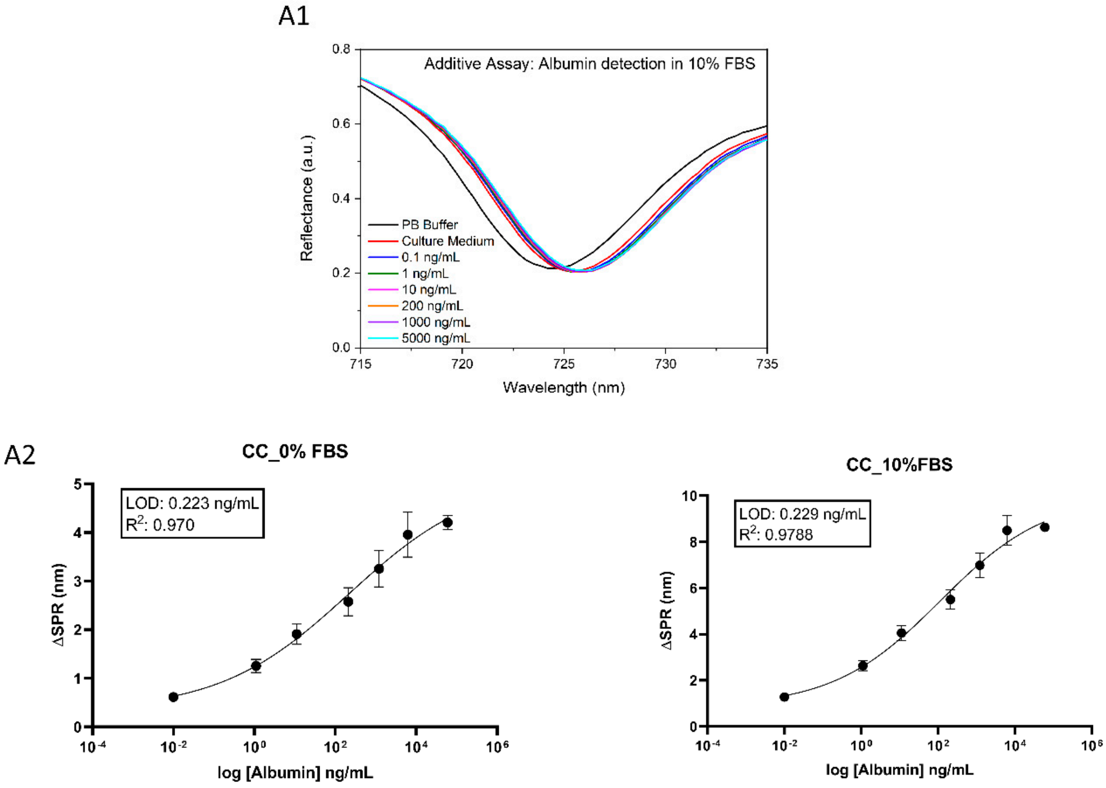

Figure S1
